# Genome-Wide Expression of MicroRNAs Is Regulated by DNA Methylation in Hepatocarcinogenesis

**DOI:** 10.1155/2015/230642

**Published:** 2015-03-11

**Authors:** Jing Shen, Shuang Wang, Abby B. Siegel, Helen Remotti, Qiao Wang, Iryna Sirosh, Regina M. Santella

**Affiliations:** ^1^Department of Environmental Health Sciences, Mailman School of Public Health, Columbia University Medical Center, New York, NY 10032, USA; ^2^Herbert Irving Comprehensive Cancer Center, Columbia University Medical Center, New York, NY 10032, USA; ^3^Department of Biostatistics, Mailman School of Public Health, Columbia University Medical Center, New York, NY 10032, USA; ^4^Department of Medicine, Columbia University Medical Center, New York, NY 10032, USA; ^5^Department of Pathology and Cell Biology, Columbia University Medical Center, New York, NY 10032, USA

## Abstract

*Background.* Previous studies, including ours, have examined the regulation of microRNAs (miRNAs) by DNA methylation, but whether this regulation occurs at a genome-wide level in hepatocellular carcinoma (HCC) is unclear. *Subjects/Methods.* Using a two-phase study design, we conducted genome-wide screening for DNA methylation and miRNA expression to explore the potential role of methylation alterations in miRNAs regulation. *Results.* We found that expressions of 25 miRNAs were statistically significantly different between tumor and nontumor tissues and perfectly differentiated HCC tumor from nontumor. Six miRNAs were overexpressed, and 19 were repressed in tumors. Among 133 miRNAs with inverse correlations between methylation and expression, 8 miRNAs (6%) showed statistically significant differences in expression between tumor and nontumor tissues. Six miRNAs were validated in 56 additional paired HCC tissues, and significant inverse correlations were observed for miR-125b and miR-199a, which is consistent with the inactive chromatin pattern found in HepG2 cells. *Conclusion.* These data suggest that the expressions of miR-125b and miR-199a are dramatically regulated by DNA hypermethylation that plays a key role in hepatocarcinogenesis.

## 1. Introduction

MicroRNAs (miRNAs) are a class of endogenous, short, single-stranded RNAs which help regulate gene expression; they contribute to a variety of physiologic processes, such as proliferation, differentiation, and apoptosis [1]. Recent studies have shown that the expression levels of miRNAs are primarily downregulated in hepatocellular carcinoma (HCC) tumor tissues compared with adjacent nontumor/cirrhotic or normal liver tissues, implicating a tumor suppressive role for miRNAs in hepatocarcinogenesis (reviewed in [1, 2]). Altered DNA methylation in miRNA host genes has been frequently associated with abnormal miRNA expression in animal models and cancer cell lines [3, 4], indicating potential epigenetic mechanisms for their regulation. Previous studies, including ours, examined whether the aberrant expression of miRNAs in human HCC may be regulated by DNA methylation alterations. A few host genes of miRNAs (miR-1-1, miR-10a, miR-122, miR-124, miR-129-2, miR-137, miR-203, miR-335, miR-503, miR-517a, miR-517c, and miR-520e) are consistently hypermethylated in HCC tumor tissue [3, 5]. However, the associations of expression and methylation status for many tumor suppressive miRNAs are still unknown, especially for those specifically expressed in liver tissues (e.g., miR-22, miR-122, miR-125b, miR-152, miR-194, miR-199, and miR-215). Thus, whether this epigenetic mechanism commonly occurs at a genome-wide level in hepatocarcinogenesis is largely unknown, limiting interpretation of dysregulated miRNAs and their potential application as diagnostic or therapeutic targets.

Using a two-phase study design, we conducted a genome-wide screen to analyze miRNA expression profiles and DNA methylation in a cross-sectional study of HCC tumors and adjacent nontumor tissues. By comprehensively examining the correlations between DNA methylation and miRNA expression profiles at a genomic level, we hoped to identify the most abundant changes in miRNA expression in tumor tissue that are regulated by aberrant DNA methylation, which may have clinical significance.

## 2. Methods

### 2.1. Study Subjects and Biospecimens

This study was approved by the Institutional Review Board of Columbia University Medical Center (CUMC). One hundred and thirty-two frozen HCC tissues from 66 patients were collected by the Center for Liver Disease and Transplantation and stored in the Molecular Pathology Shared Resource of the Herbert Irving Comprehensive Cancer Center (HICCC). Histological evaluation of hematoxylin and eosin (H.E.) stained 4 micron thick sections of frozen liver tumor and adjacent nontumor tissues stored at −20°C included assessment of the presence, viability, and percent tumor. Tumor samples were macrodissected to ensure >80% purity of tumor. To insure the DNA/RNA extracted from adjacent normal tissue did not contain tumor cells, tissue sections were cut from frozen tissues and H.E. stained. The stained sections were reviewed by the study pathologist (H.R.) to ensure no tumor tissues or cells were present [6]. Tumor stage was determined according to the American Joint Committee on Cancer (AJCC) criteria [7]. Then several sections were cut from the same tissues for DNA/RNA extraction. Adjacent tissues were also evaluated with respect to the presence (Batts-Ludwig stage of 4) or absence of cirrhosis (Batts-Ludwig stage < 4). Information on clinicopathological features including *α*-fetoprotein levels, tumor size and number, tumor grade, presence of vascular invasion, and capsular infiltration was obtained from medical records. HBV (HBsAg) and HCV (anti-HCV) status, determined by immunoassay, were obtained from medical records. Ten paired HCC tumor/adjacent nontumor tissues were randomly selected as the discovery set to evaluate miRNA expression profiles. The remaining 56 pairs were used as a validation set to test candidate miRNAs.

### 2.2. Laboratory Methods

Total RNA, including miRNAs, was isolated from 66 frozen HCC tumor and 66 adjacent nontumor tissues by RNeasy Microarray Tissue Mini Kits (Qiagen, Frederick, MA) according to the manufacturer's protocol. TaqMan Low Density Arrays (TLDA, Applied Biosystems, Foster City, CA), covering 733 miRNAs (670 unique human mature miRNAs), were used to generate genome-wide miRNA profiles for the discovery set and data were then deposited in NCBI's Gene Expression Omnibus (GEO) database (accession number GSE54751) [8]. TaqMan MicroRNA assays were used to measure expression of 6 candidate miRNAs in the discovery set as well as the validation set. U6 snRNA stable by liver tumor/adjacent tissue status (Ct: 21.19 versus 21.08, *P* = 0.398) was used as an endogenous control to normalize the relative expression of target miRNAs using the 2^(−ΔΔCt)^ approach [9].

DNA was extracted from the tumor/adjacent nontumor tissues by standard proteinase K/RNase treatment and phenol/chloroform extraction. Bisulfite modification of 1 *μ*g DNA was conducted using an EZ DNA Methylation Kit (Zymo Research, Irvine, CA) according to the manufacturer's procedure. The Infinium Methylation 450 K assay was performed according to Illumina's standard protocol as reported previously [6]. The 450 K array includes 3,439 CpG sites covering 727 human miRNAs and these data were used for further analysis. Methylation levels of CpG sites were calculated as beta-values (*β* = [intensity of the methylated allele (M)/(intensity of the unmethylated allele (U) + intensity of the methylated allele (M))] × 100) [10]. For quality control (QC), methylation measures with a detection *P* value > 0.05 and samples with a CpG coverage < 95% were removed. The complete methylation profiles have been deposited in NCBI's GEO database and are available through series accession number GSE54503 [8].

### 2.3. Integrative Analyses with the Encyclopedia of DNA Elements (ENCODE) and Oncomine Data

ENCODE data for human hepatoblastoma cell line (HepG2) and the seven other cancer cell lines (GM12878, H1-hESC, HSMM, HUVEC, K562, NHEK, and NHLF) were incorporated with DNA methylation results from miRNA host genes to examine the cooperative role of histone modifications and deoxyribonuclease (DNase I) hypersensitivity in chromatin activity (https://genome.ucsc.edu/ENCODE/). Previous results suggest that H3K4me1 (monomethylation of lysine 4) is associated with active chromatin outside of promoters (e.g., enhancers); H3K4me3 (trimethylation of lysine 4) is primarily associated with active promoters; H3K27ac (acetylation of lysine 27) is associated with both active promoters and enhancers [11, 12]; and DNase I hypersensitive sites are associated with active histone marks and transcription factor binding [13]. The Oncomine database (https://www.oncomine.org) [14, 15] that includes cancer microarray data deposited in GEO and the Stanford Microarray Database (SMD) were used to determine expression of mRNAs of miRNA host or target genes in HCC tumor and/or precursor/normal liver tissues [6, 16]. The gene expression data were log_2_ transformed and median-centered per array, and the standard deviation (SD) was normalized to one per array [14, 15].

## 3. Statistics

We explored genome-wide miRNA profiles in HCC tissues by the univariate test and Benjamini-Hochberg false discovery rate (FDR) adjustment using Limma [17, 18] to identify aberrant expression of miRNAs. Expression levels of miRNAs in different tissues (tumor or nontumor) were centered and distances were determined using the uncentered Pearson correlation coefficient. The average linkage hierarchical clustering was performed with Cluster 3.0 [19, 20] and displayed with Java Treeview [20, 21] based on significant miRNAs (FDR < 0.05). Spearman correlation coefficients were calculated to evaluate the relationships between miRNA expression and DNA methylation alterations. Paired *t*-tests with Bonferroni correction for multiple testing were used to identify miRNA host genes' CpG sites that are differentially methylated between tumor and adjacent nontumor tissues. A significant difference was defined as a CpG site with a Bonferroni-corrected *P* value ≤ 0.05 which corresponded to a raw *P* value of ≤1.45 × 10^−5^. All statistical analyses were conducted using the R language (http://www.r-project.org/) and Statistical Analysis System  9.0 (SAS Institute, Cary, NC).

## 4. Results

### 4.1. Clinical and Pathological Characteristics of HCC Patients

Comparisons of clinical and pathological characteristics for HCC patients in the discovery and validation sets are shown in Supplementary Table S1 in Supplementary Material available online at http://dx.doi.org/10.1155/2015/230642. The mean ages and the proportions of ethnicity, HBV/HCV postive, cigarette smokers, and alcohol drinkers were similar. Because we selected 5 male and 5 female patients in the discovery set to compare miRNA differences by gender, the proportion of females in discovery set was different from the validation set with fewer female HCC cases in the validation set (*P* = 0.039). There were more patients with hepatic resection in the discovery set and more transplant patients in the validation set (*P* < 0.001). No statistically significant differences were observed for other clinical characteristics in the discovery and validation sets, including AFP levels, tumor size, grade, cirrhosis, and survival outcome, indicating the overall comparability of the two sets.

### 4.2. Aberrant miRNA Expression Profiles Identified in HCC Tumor Tissue

**Figure 1:**
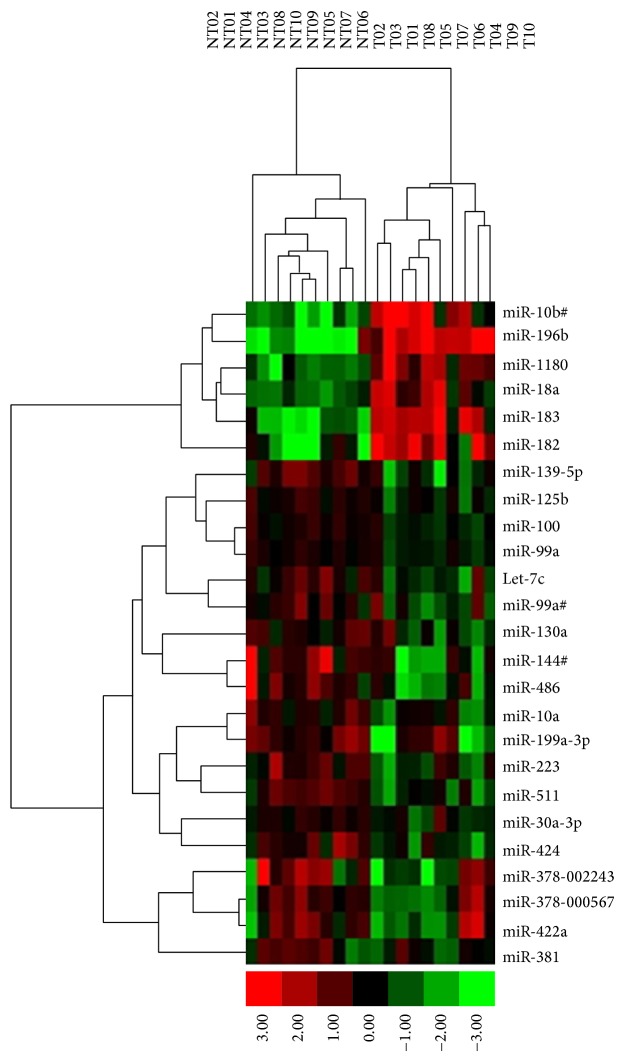
Hierarchical cluster analyses of 25 dsyregulated miRNAs that differentiate 10 HCC tumor tissues from 10 adjacent nontumor tissues. T represents tumor tissue, and NT represents adjacent nontumor tissue. Red indicates overexpression and green downregulation.

After adjusting for multiple comparisons using the FDR, we observed 25 miRNAs were significantly dysregulated in HCC tumor tissues (FDR < 0.05) with upregulation of 6 miRNAs and downregulation of 19 miRNAs (Table 1). The fold changes of the overexpressed miRNAs ranged from 2.6-fold for miR-18a to 18-fold for miR-196b. The fold changes for downregulated miRNAs ranged from −2.5-fold for miR-99a to −7.5-fold for miR-144^#^. A few miRNAs (miR-139, miR-381, miR-486, and miR-1180) were identified for the first time as aberrantly expressed in HCC tumor tissues. Comparisons of several over- or underexpressed miRNAs are displayed in Supplementary Figure S1. The cluster diagram and heatmap with the 25 significantly aberrant miRNAs show a perfect differentiation of the HCC tumors from nontumor tissues (Figure 1), suggesting their potential clinical application as HCC diagnostic biomarkers.

### 4.3. DNA Methylation Alterations in HCC Tumor Tissue

We compared DNA methylation levels of 3,439 miRNA-relevant CpG sites in 10 pairs of HCC tumor and nontumor tissues in the discovery set. After Bonferroni adjustment, a total of 28 miRNA CpG sites significantly differed in DNA methylation between tumor and adjacent nontumor tissues (Supplementary Table S2). Overall, the predominant DNA methylation change occurring in HCC tumors was demethylation. The 28 significant CpG sites clearly distinguished tumor from nontumor tissue without misclassification (Supplementary Figure S2). We validated DNA methylation for those CpG sites in additional 56 pairs of tumor and nontumor tissues and found 100% consistent results (Supplementary Table S3).

### 4.4. DNA Methylation and Relevant miRNAs Expression

We further analyzed the associations between DNA methylation of 1,515 CpG sites and the expression of the 222 relevant miRNAs covered by both TLDA and 450 K arrays and detectable in at least 80% of samples. Supplementary Figure S3 reveals that a total of 1,014 CpG sites were hypomethylated, and 501 CpG sites were hypermethylated. An inverse correlation between DNA methylation and miRNA expression was observed for 133 miRNAs, which included 55 (92%) upregulated miRNAs and 78 (48%) downregulated miRNAs (data not shown). Among the 133 miRNAs showing inverse correlation patterns between methylation and miRNA expression, 8 miRNAs (miR-10a, miR-18a, miR-125b, miR-130a, miR-144, miR-182, miR-199a, and miR-1180) were significantly different in expression levels between tumor and nontumor tissues in the discovery set (Table 1). The Spearman rank correlation between methylation and expression was statistically significant for four miRNAs (mir-125b-1, mir-144, mir-199a-1, and mir-1180). Only for mir-125b-1 and mir-199a-1 were there significant differences in DNA methylation levels at a raw *P* value of 0.05; none were significant after adjusting for multiple comparisons (data not shown).

### 4.5. Validation of the Inverse Methylation/Expression Pattern

We validated the expression levels for 6 miRNAs (miR-10a-5p, miR-18a-5p, miR-125b-5p, miR-182, miR-199a-3p, and miR-1180) with inverse methylation/expression correlations. Consistent over- or underexpression in tumor tissues was observed for all 6 miRNAs in both the discovery and validations sets (Table 3). miR-10a-5p, miR-125b-5p, and miR-199a-3p were consistently repressed, while miR-18a-5p, miR-182, and miR-1180 were overexpressed in tumor tissues. Statistically significant differences were observed for 5 miRNAs that were aberrantly expressed in HCC tumor tissues in the validation set. The fold changes varied from −2.58-fold for miR-199a-3p to 2.45-fold for miR-182 (Table 3). The expression of miR-18a-5p revealed no significant difference between tumor and nontumor tissues (*P* = 0.115) but became significant after combination of the validation and discovery sets (*P* = 0.016).

Inverse Spearman rank correlations between methylation and expression were observed for mir-18a, mir-125b-1, mir-182, mir-199a-1, and mir-1180; mir-125b-1 and mir-199a-1 achieved statistical significance (Supplementary Table S4). The correlation coefficients ranged from −0.23 to −0.63. Eighty-four percent of subjects (47/56) displayed inverse methylation and expression patterns for miR-125b or miR-199a. These results verified the findings from the discovery set that DNA hypermethylation potentially plays a regulatory role in the repression of miR-125b and miR-199a expression. The expression levels of miR-125b were, respectively, −1.15 and −1.45 (*P* = 0.21) for HBV negative or positive tissues and −1.19 and −1.09 (*P* = 0.72) for HCV negative or positive tissues. The expression levels of miR-199a were, respectively, −1.98 and −2.51 (*P* = 0.19) for HBV negative or positive tissues and −2.41 and −1.58 (*P* = 0.075) for HCV negative or positive tissues (data not shown).

### 4.6. Integrative Analyses with ENCODE and Oncomine Data

**Figure 2:**
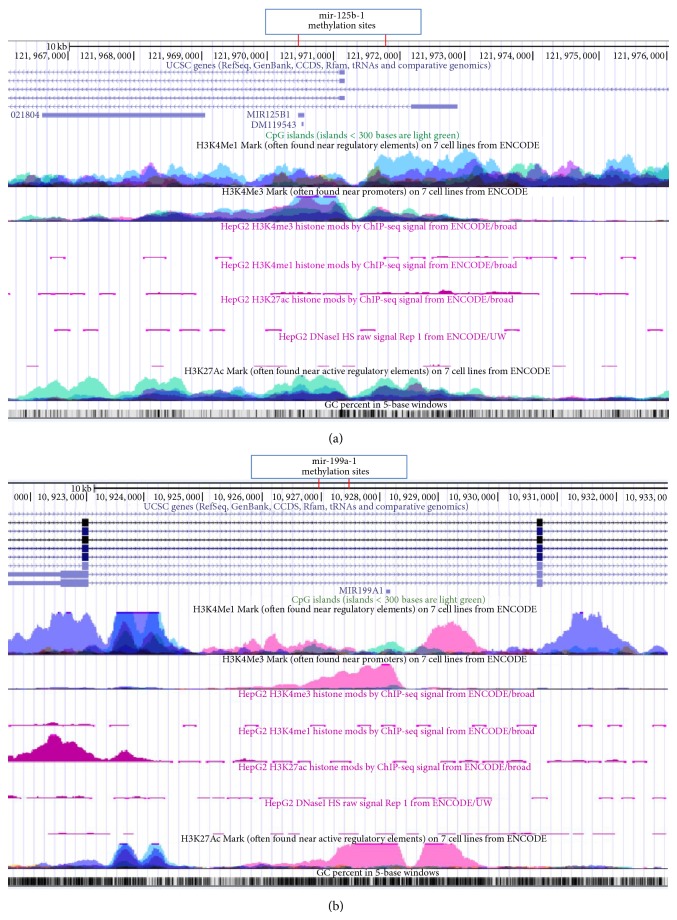
UCSC genome browser tracks showing histone modification (H3K4me1, H3K4me3, and H3K27ac) and DNase I cleavage states around the hypermethylated CpG sites of mir-125b-1 and mir-199a-1 in the HepG2 cell line and in seven other cancer cell lines. The genome browser map from top to bottom is the CpG island; layered H3K4Me1 and H3K4Me3 marks in the seven other cancer cell lines; H3K4Me3, H3K4Me1, and H3K27Ac marks and DNase I hypersensitive sites in HepG2 cells; H3K27Ac activator in seven cancer cell lines; and GC percent. (a) Genomic region around mir-125b-1 (chr11:121,962,000-121,976,000). Consistent with DNA hypermethylation and underexpression of miR-125b, no active histone marks (H3K4me1, H3K4me3, and H3K27ac) and DNase I hypersensitive sites were observed in HepG2 cells, but higher levels of H3K4me1, H3K4me3, and H3K27ac were found at the same region in the seven other cancer cell lines. (b) The genomic region around mir-199a-1 (chr19:10,917,000-10,933,000). Consistent with DNA hypermethylation and underexpression of miR-199a, there was no activation of histone markers (H3K4me1, H3K4me3, and H3K27ac), as well as closed chromatin (no peak for DNase I hypersensitive sites) in HepG2 cells, which is different from the pattern observed in the other cancer cell lines (showing high to intermediate peaks for H3K4me1, H3K4me3, and H3K27ac marks).

We integrated ENCODE data with DNA hypermethylation results for mir-125b-1 and mir-199a-1 in HepG2 and seven other cancer cell lines to examine the cooperative role of histone modifications and DNase I hypersensitivity in chromatin activity (Figure 2). No active histone marks (H3K4me1, H3K4me3, and H3K27ac) or DNase I hypersensitive sites were observed in HepG2 cells around the mir-125b-1 (Figure 2(a)) hypermethylated CpG sites (closed chromatin). Higher levels of H3K4me1, H3K4me3, and H3K27ac were found in the seven other cancer cell lines in the same region (open chromatin). Similarly, no signature of active histone marks (H3K4me1, H3K4me3, and H3K27ac) or active regulation regions (DNase I hypersensitive sites) was found around the hypermethylated region of mir-199a-1 in HepG2 cells (Figure 2(b)). High to intermediate levels of active histone marks (H3K4me1, H3K4me3, and H3K27ac) were found in the seven other cancer cell lines, suggesting active chromatin. These results indicate a potential cooperative role for DNA hypermethylation and histone modifications in the repression of mir-125b-1 and mir-199a-1, especially in the HepG2 cell line.

mir-199a-1 is located on chromosome 19p13.2, in intron 16 of the host gene* DNM2* (Dynamin 2). Integrating results with Oncomine data, we found miR-199a was cosuppressed or coexpressed with* DNM2* mRNA; that is, significant underexpression of* DNM2* (log_2_ median-centered intensity) was observed in HCC tumor (0.675) compared with precursor (0.717) and normal (0.870) liver tissues (Supplementary Figure S4), which is consistent with the underexpression of miR-199a in HCC tumor tissue. In contrast, the mRNA levels of miRNAs' target genes were significantly increased in HCC tumor tissues compared with precursor and normal liver tissues and were inversely associated with the expression of tumor suppressive miRNAs. For miR-125b, overexpression of the target genes in HCC tumor tissues has been found for B-Cell CLL/Lymphoma 2 (*BCL2*), v-erb-b2 avian erythroblastic leukemia viral oncogene homolog 2/3 (*ERBB2*/*3*), sirtuin7 (*SIRT7*), v-ets avian erythroblastosis virus E26 oncogene homolog 1 (*ETS1*), myeloid cell leukemia sequence 1 (*Mcl-1*), interleukin 6 receptor (*IL6R*), and Lin-28 Homolog B (*LIN28B*). Expression of one target gene,* ERManI* (endoplasmic reticulum mannosidase I) is unknown due to lack of Oncomine data. For miR-199a, several target genes were analyzed, including mammalian target of rapamycin (*mTOR*),* Met* prooncogene,* HIF-1α* , clathrin heavy chain (*CHC*), discoidin domain receptor-1 (*DDR1*), and* CD44*. Overexpressed* mTOR*,* Met*,* CHC*, and* DDR1* were verified in HCC tumor tissues, as compared with precursor and normal liver tissues. No significant dysregulation was observed for the target genes* CD44* and* HIF-1α* . Supplementary Figure S5 displays two representative target genes for miR-125b (*LIN28B*) and miR-199a (*mTOR*) that were overexpressed in HCC tumor tissues.

## 5. Discussion

Emerging evidence, including the current study, suggests that miRNA deregulation contributes to HCC development. After adjusting for false discovery rates, we found 25 miRNAs significantly dysregulated in HCC tumor tissues (Table 1); they could differentiate tumor from nontumor tissues without misclassification (Figure 1). Several miRNAs (miR-139, miR-381, miR-486, and miR-1180) were identified for the first time as aberrantly expressed in HCC tumor tissue. Through genome-wide screening for DNA methylation and miRNA expression and integrative analyses with ENCODE data, 133 miRNAs showed inverse correlation patterns between methylation and expression. Only 8 miRNA (6%) expression levels were significantly different between tumor and nontumor tissues in the discovery set (Table 1). Even fewer miRNAs (mir-125b-1, mir-144, mir-199a-1, and mir-1180) had significant inverse methylation and expression correlations (Table 2). With validation in 56 additional paired HCC tissues, statistically significant differences were observed for 5 miRNAs that were aberrantly expressed in HCC tumor tissues (Table 3). Significant inverse correlations were verified only for mir-125b-1 and mir-199a-1. The correlation coefficients ranged from −0.23 to −0.63 (Supplementary Table S4). This regulation pattern was supported by the lower levels of active histone marks (H3K4me1, H3K4me3, and H3K27ac) and DNase I hypersensitive sites that lead to closed chromatin specifically in HepG2 cells. In seven other cancer cell lines, no cooperative histone marks were observed (Figure 2). These results suggest that DNA hypermethylation, in collaboration with histone modifications, play a crucial regulatory role in the repression of miR-125b and miR-199a as tumor suppressors in hepatocarcinogenesis. This is being clinically investigated with demethylating agents and histone deacetylase (HDAC) inhibitors in HCC [22, 23].

mir-125b-1 is located on chromosome 11q24.1, where no coding gene is nearby except* BLID* (BH3-like motif-containing cell death inducer) situated 16-kb away. Previous studies found that expression levels of miR-125b were generally downregulated in HCC tissues [24–29] as well as in other cancers, such as prostate, breast, ovarian, and thyroid anaplastic carcinomas, as compared with nontumor tissues. Importantly, ectopic expression of miR-125b inhibited the cell growth, proliferation, migration, invasion, and tumorigenesis of cancer cells [27, 30–32], suggesting its tumor suppressive role. Thus far, only one study found that promoter hypermethylation of mir-125b-1 partially accounted for the reduction of miR-125b expression in breast cancer [33]. For the first time, we found a significant inverse correlation between mir-125b-1 hypermethylation and relevant miR-125b underexpression in HCC, which is consistent with closed chromatin (low levels of active histone marks H3K4me1, H3K4me3, and H3K27ac). The biological functions of miR-125b are mainly achieved by its repression of target prooncogenes or oncogenes, such as* BCL2*,* ERBB2*/*3*,* ERManI*,* SIRT7*,* ETS1*,* Mcl-1*,* IL6R*, and* LIN28B*. Evaluating with Oncomine data, we found most target genes (*BCL2*,* ERBB2*/*3*,* SIRT7*,* ETS1*,* Mcl-1*,* IL6R*, and* LIN28B*) were significantly activated in HCC tumor tissues, consistent with miR-125b underexpression. This reverse correlation further verified the important regulatory role of DNA hypermethylation in miR-125b underexpression.

The expression of miR-199a is diversely deregulated in many types of cancer. It is significantly downregulated in breast [34], bladder [35], ovarian [36], and testicular germ cell tumor (TGCT) [37] but upregulated in cervical [38], colorectal [39], gastric cancer [40], and hepatoblastoma [41]. Moreover, overexpression of miR-199a was considered a signature for poor cancer prognosis and a contributor to more advanced lymphatic invasion, lymph node metastasis, and late TNM stage in colorectal and gastric cancer [39, 40]. However, significant downregulation of miR-199a has been consistently observed in human HCC cell lines as well as in tumor tissues. DNA hypermethylation has been found to reduce miR-199a expression in TGCT [37], and silencing of DNMT1 with siRNA or treatment with 5-azaC can restore the expression of miR-199a [42]. However, this methylation change occurred within a genomic region of* DNM3* (1q24.3) that encodes mir-199a-2—another precursor of miR-199a. DNA hypermethylation was only found in mir-199a-1 within the host gene of* DNM2* (19p13.2), but not in* DNM3* (data not shown). The expression of miR-199a is usually coordinately expressed with its host gene (*DNM2*), which was confirmed by our results (Supplementary Figure S4). No aberrant expression of DNM3 was obtained in HCC tumor tissue. In contrast, expression levels of miR-199a should be inversely associated with its target genes in HCC, such as mTOR,* Met* prooncogene,* HIF-1α* ,* CHC*,* DDR1*, and* CD44*. Our integrative analyses also verified the overexpression of four target genes (*mTOR*,* Met*,* CHC*, and* DDR1*) in HCC tumor tissue, although no significant differences were observed for targets of CD44 and HIF-1*α*. These data suggest that DNA hypermethylation specifically of mir-199a-1 precursor (19p13.2), but not the precursor of mir-199a-2 (1q24.3), is important for miR-199a regulation, as well as subsequent activation of downstream target genes in hepatocarcinogenesis. The inhibitors of Met and mTOR (the target genes of miR-199a) are in trial development for HCC [43–45]. The most clinically significant effects of Tivantinib, an inhibitor of Met, were observed in a subgroup of HCC patients with high Met expression in terms of time to progression and overall survival [44], which suggests a better response for the therapy by inhibition of the relevant pathway. These data provide additional evidence to support the crucial regulatory role of miR-199a in HCC. Reactive oxygen species (ROS) induced methylation may partially explain our observations in HCC. ROS can increase promoter methylation of mir-125b-1 and mir-199a-1 by recruiting DNA methyltransferase 1 (DNMT1) to oxidative stress-induced damaged chromatin before the DNA is repaired, which leads to overexpression of target genes (*ERBB2* and* ERBB3*) for both miR-125b and miR-199a* in vitro* and* in vivo* [46].

The strengths of the current study include the use of genome-wide arrays for both DNA methylation and miRNA expression profiles, which provide comprehensive data for identification of miRNAs epigenetically regulated by DNA methylation. The two-phase study design and large sample size allow us to validate the correlation patterns of DNA methylation and miRNA expression with sufficient statistical power. Integrative analyses with ENCODE and Oncomine data help us to better understand the connection between DNA methylation and histone modification, as well as miRNA and mRNA expression of host and/or target genes. One limitation is that the numbers of miRNAs covered by the two arrays are different (Supplementary Figure S3). For instance, two significant dysregulated miRNAs (miR-378 and miR-422a) on the TLDA array were not covered by the Infinium Methylation 450 K arrays. Therefore, we were unable to clarify the role of methylation in regulation of these two miRNAs. Secondly, a positive correlation of DNA methylation and miRNA expression pattern was observed for several miRNAs, indicating additional biological regulatory mechanisms are involved that need further exploration. Because the ENCODE data did not include HCC cell lines (SNU-449, JHH2), we conducted the integrative analyses using HepG2 cells derived from a human hepatoblastoma. Therefore, we acknowledge that the relevant DNA methylation and miRNAs expression patterns may be different for the two types of cell lines, and the results should be interpreted with caution.

## 6. Conclusion

In summary, we found two miRNAs (miR-125b and miR-199a) are mainly regulated by DNA hypermethylation, supporting their tumor suppressor role in the repression of downstream target oncogenes. This result provides additional insight into the etiological role of epigenetic change in hepatocarcinogenesis. Further studies attempting to directly restore miRNA expression and/or indirectly modify DNA methylation are in progress to control this aggressive tumor that is currently increasing in the US population [47].

## Supplementary Material

The supplementary material contains basic clinical and pathological information for HCC cases (Supplementary Table S1), and the aberrant DNA methylation markers examined in discovery set (Supplementary Table S2) and validation set (Supplementary Table S3). It contains the validation results of the inverse correlations between DNA methylation differences and miRNAs fold changes (Supplementary Table S4), and the comparison of miRNAs expression between tumor and non-tumor tissues (Supplementary Figure S1), and the hierarchical cluster of DNA methylation between tumor and adjacent non-tumor tissues in the discovery set (Supplementary Figure S2). The supplementary material also contains a Venn diagram to describe the number of miRNAs covered by both TLDA and 450K arrays (Supplementary Figure S3), and the comparisons of miRNA host gene (Supplementary Figure S4) and target genes' (Supplementary Figure S5) expression in HCC tumor, precursor and normal liver tissues.

## Figures and Tables

**Table 1:** Fold change of top 25 significant miRNAs^*^ in the discovery set (*N* = 10 pairs).

miRNAs^†^	Tumor	Nontumor	Fold change^**^	*P* values
miR-196b	−8.94 (1.17)	−13.38 (1.50)	18.00	2.07 · *E* − 05
miR-10b^#^	−10.01 (1.92)	−13.16 (1.83)	8.94	1.90 · *E* − 04
miR-182	−10.83 (1.68)	−13.45 (2.72)	8.34	9.15 · *E* − 04
miR-183	−13.03 (1.31)	−15.00 (1.66)	8.00	1.43 · *E* − 04
miR-1180	−10.25 (1.01)	−12.13 (1.16)	3.73	6.01 · *E* − 04
miR-18a	−7.74 (1.33)	−9.10 (0.78)	2.57	2.47 · *E* − 03
miR-99a^#^	−10.87 (0.98)	−9.27 (0.90)	−2.50	3.09 · *E* − 03
miR-30a-3p	−5.17 (0.86)	−3.84 (0.69)	−2.51	2.44 · *E* − 03
miR-381	−14.24 (0.85)	−12.89 (1.17)	−2.55	3.76 · *E* − 03
miR-100	−5.24 (0.76)	−3.83 (0.88)	−2.66	1.60 · *E* − 03
miR-125b	−4.97 (1.04)	−3.44 (0.88)	−2.89	3.46 · *E* − 03
miR-99a	−5.57 (0.74)	−4.26 (0.83)	−3.01	2.12 · *E* − 03
miR-378-002243^‡^	−4.69 (1.14)	−3.03 (0.84)	−3.14	2.38 · *E* − 03
miR-130a	−9.39 (1.02)	−7.72 (1.04)	−3.18	1.34 · *E* − 03
miR-422a^†^	−8.72 (1.42)	−7.04 (1.02)	−3.20	2.18 · *E* − 03
let-7c	−8.62 (1.08)	−6.88 (0.50)	−3.32	5.15 · *E* − 04
miR-10a	−8.30 (1.37)	−6.45 (1.21)	−3.58	2.76 · *E* − 03
miR-223	−1.79 (1.36)	0.18 (0.79)	−3.89	1.38 · *E* − 03
miR-424	−11.84 (1.07)	−9.86 (1.21)	−3.94	4.60 · *E* − 05
miR-511	−11.34 (1.16)	−9.15 (0.68)	−4.56	8.14 · *E* − 05
miR-139-5p	−7.00 (1.37)	−4.76 (0.94)	−4.69	1.20 · *E* − 03
miR-199a-3p	−5.27 (2.45)	−2.68 (1.14)	−6.02	2.11 · *E* − 03
miR-486	−8.81 (1.10)	−6.21 (1.90)	−6.06	3.26 · *E* − 04
miR-378-000567^†^	−11.65 (2.30)	−8.89 (2.23)	−6.82	1.01 · *E* − 03
miR-144^#^	−9.98 (1.66)	−7.06 (1.49)	−7.52	2.53 · *E* − 04

^*^False discovery rate (FRD) <0.05; ^†^“miR-” refers to the mature miRNA; ^**^fold change >0 indicates miRNAs overexpression, <0 indicates miRNAs downexpression; ^‡^not included in the Infinium Methylation 450K assay; the miRNA following ^#^ indicates a miRNA expressed at low levels relative to the same miRNA without ^#^, which shares a pre-miRNA hairpin.

**Table 2:** Inverse correlations between DNA methylation differences and miRNA fold changes in the discovery set (*N* = 10 pairs).

mir^*^ (chromosome location)	CpG sites	Distance to miRNA (bp)	Methylation difference (T versus NT)	Fold change (T versus NT)	Spearman correlation coefficients	*P* values
mir-10a 17q21.32 46,657,200–46,657,309	cg15649236	195	0.25	−3.58	−0.35	1.32 · *E* − 01
	cg01572694	246	0.19	−3.58	−0.33	1.50 · *E* − 01
	cg14884929	347	0.10	−3.58	−0.09	7.19 · *E* − 01

mir-18a 13q31.3 (92,002,997–92,003,088)	cg17799287	1241	−0.09	2.57	−0.26	2.71 · *E* − 01
	cg07641807	1046	−0.09	2.57	−0.17	4.78 · *E* − 01
	cg23665802	667	−0.11	2.57	−0.27	2.46 · *E* − 01
	cg02297838	551	−0.15	2.57	−0.24	3.14 · *E* − 01

mir-125b-1 11q24.1 121,970,465–121,970,552	cg02101355	142	0.06	−2.89	−0.69	8.00 · **E** − 04
	cg03891346	173	0.04	−2.89	−0.74	2.00 · **E** − 04
	cg24150623	540	0.21	−2.89	−0.74	2.00 · **E** − 04
	cg06749053	667	0.12	−2.89	−0.46	3.90 · **E** − 02
	cg20475322	680	0.16	−2.89	−0.65	1.90 · **E** − 03
	cg24603444	782	0.15	−2.89	−0.62	4.30 · **E** − 03
	cg16865908	0	0.05	−2.89	−0.26	2.71 · *E* − 01
	cg26916936	61	0.04	−2.89	−0.17	4.74 · *E* − 01
	cg07685357	1402	0.07	−2.89	−0.34	1.46 · *E* − 01

mir-130a 11q12.1 57,408,671–57,408,759	cg16520038	11	0.10	−3.18	−0.03	8.85 · *E* − 01
	cg10512089	0	0.16	−3.18	−0.18	4.44 · *E* − 01
	cg01681881	1250	0.03	−3.18	−0.04	8.55 · *E* − 01

mir-144 17q11.2 27,188,551–27,188,636	cg19196414	0	0.001	−7.52	−0.48	3.30 · **E** − 02

mir-182 7q32.2 129,410,223–129,410,332	cg16576544	0	−0.05	8.34	−0.11	5.14 · *E* − 01
	cg24423782	85	−0.05	8.34	−0.09	7.10 · *E* − 01
	cg17677032	95	−0.03	8.34	−0.08	7.37 · *E* − 01
	cg04579608	103	−0.05	8.34	−0.04	8.87 · *E* − 01
	cg13713066	325	−0.03	8.34	−0.07	7.81 · *E* − 01

mir-199a-1 19p13.2 10,928,102–10,928,172	cg27648270	0	0.09	−6.02	−0.78	1.00 · **E** − 05
	cg18544365	6	0.08	−6.02	−0.66	1.70 · **E** − 03
	cg23047544	39	0.09	−6.02	−0.73	4.00 · **E** − 04
	cg02660440	61	0.09	−6.02	−0.75	1.00 · **E** − 04
	cg06754197	150	0.07	−6.02	−0.83	1.00 · **E** − 05
	cg02907064	155	0.06	−6.02	−0.74	2.00 · **E** − 04
	cg23068797	377	0.10	−6.02	−0.77	1.00 · **E** − 05
	cg03216043	467	0.10	−6.02	−0.72	3.00 · **E** − 04
	cg13965612	524	0.07	−6.02	−0.50	2.45 · **E** − 02

mir-1180	cg04864152	15	−0.08	3.73	−0.05	8.25 · *E* − 01
	cg20272287	18	−0.10	3.73	−0.01	9.60 · *E* − 01
	cg02796621	678	−0.05	3.73	−0.05	8.25 · *E* − 01
	cg02206323	1181	−0.05	3.73	−0.49	3.06 · **E** − 02
	cg26619894	1277	−0.04	3.73	−0.27	2.67 · **E** − 03

^*^“mir-” refers to the precursor miRNA (pre-miRNA).

**Table 3:** Validation of six miRNAs' expression levels by qRT-PCR assays in the discovery (*N* = 10 pairs) and validation (*N* = 56 pairs) sets.

miRNAs	log_2_ expression levels		Fold change^*^	*P *
	Mean (SD)			
	Tumor	Nontumor		
Discovery set				
miR-10a-5p	−2.77 (1.03)	−2.52 (0.91)	−1.19	4.50 · *E* − 01
miR-125b-5p	−1.45 (1.34)	−1.13 (1.05)	−1.25	3.83 · *E* − 01
miR-199a-3p	−2.39 (2.16)	−1.67 (1.27)	−1.64	2.71 · *E* − 01
miR-18a-5p	−7.20 (1.58)	−8.49 (0.84)	2.45	1.39 · **E** − 02
miR-182	−8.63 (1.93)	−10.62 (1.89)	3.97	6.10 · *E* − 02
miR-1180	−8.21 (1.82)	−9.12 (1.06)	1.88	7.74 · *E* − 02
Validation set				
miR-10a-5p	−2.58 (1.17)	−2.04 (1.01)	−1.45	2.91 · **E** − 03
miR-125b-5p	−1.44 (1.27)	−0.85 (1.10)	−1.51	8.98 · **E** − 03
miR-199a-3p	−2.69 (2.30)	−1.32 (1.47)	−2.58	3.60 · **E** − 04
miR-18a-5p	−8.09 (1.31)	−8.44 (0.95)	1.27	1.15 · *E* − 01
miR-182	−10.04 (2.03)	−11.33 (1.73)	2.45	1.15 · **E** − 03
miR-1180	−8.63 (1.60)	−9.28 (1.15)	1.57	3.54 · **E** − 03

^*^Fold change >0 indicates overexpression; <0 indicates decreased expression.
