# Supplementary material for: Genome-Wide Expression of MicroRNAs Is Regulated by DNA Methylation in Hepatocarcinogenesis
Source: Gastroenterol Res Pract. 2015 Mar 11;2015:230642. doi: 10.1155/2015/230642 (PMC4377534; doi:10.1155/2015/230642)
Supplement: Supplementary file 1 — The supplementary material contains basic clinical and pathological information for HCC cases (Supplementary Table S1), and the aberrant DNA methylation markers examined in discovery set (Supplementary Table S2) and validation set (Supplementary Table S3). It contains the validation results of the inverse correlations between DNA methylation differences and miRNAs fold changes (Supplementary Table S4), and the comparison of miRNAs expression between tumor and non-tumor tissues (Supplementary Figure S1), and the hierarchical cluster of DNA methylation between tumor and adjacent non-tumor tissues in the discovery set (Supplementary Figure S2). The supplementary material also contains a Venn diagram to describe the number of miRNAs covered by both TLDA and 450K arrays (Supplementary Figure S3), and the comparisons of miRNA host gene (Supplementary Figure S4) and target genes' (Supplementary Figure S5) expression in HCC tumor, precursor and normal liver tissues. [file 230642.f1.pdf]

Genome-Wide Expression of MicroRNAs is Regulated by DNA Methylation in Hepatocarcinogenesis

Jing Shen,[1,2*] Shuang Wang,[3] Abby B. Siegel,[4] Helen Remotti,[5] Qiao Wang,[1] Iryna Sirosh,[1] and Regina M. Santella[1,2]

[1] *Department of Environmental Health Sciences, Mailman School of Public Health, Columbia University Medical Center, New York, NY 10032, USA;*

[2] *Herbert Irving Comprehensive Cancer Center, Columbia University Medical Center, New York, NY 10032, USA;*

[3] *Department of Biostatistics, Mailman School of Public Health, Columbia University Medical Center, New York, NY 10032, USA;*

[4] *Department of Medicine, Columbia University College of Physicians and Surgeons, New York, NY 10032, USA;*

[5] *Department of Pathology and Cell Biology, College of Physicians and Surgeons, Columbia University, New York, NY 10032, USA*

Supplementary Materials

Supplementary Figure Legend

Supplementary FIGURE S1: Comparisons of up- or under-expressed miRNAs between tumor and non-tumor tissues in the discovery set (10 pairs)

MiR-196b, miR-183, miR-10b[#] and miR-182 were significantly up-regulated, while miR-511, miR-144[#], miR-486, miR-378, miR-139-5p, and miR-199a-3p were significantly down-regulated in HCC tumor tissues.

Supplementary FIGURE S2: Hierarchical cluster analysis of the top 28 significantly differentially methylated CpG sites between 10 pairs of HCC tumor and adjacent non-tumor tissues

Blue represents tumor tissue and red adjacent non-tumor tissue. The top 28 significant CpG sites can clearly distinguish HCC tumor from adjacent tissues without misclassification.

Supplementary FIGURE S3: Genome-wide associations between DNA methylation and miRNA expression covered by TLDA and 450K arrays

A Venn diagram describes the number of miRNAs covered by each assay (TLDA and 450K array), as well as detectable in over 80% samples. There are 628 miRNAs covered by both TLDA and 450K arrays, but only 222 were detectable in over 80% of samples. We focused our analyses on these 222 miRNAs. A total of 1,014 CpG sites were hypomethylated covering 206 miRNAs, and 501 CpG sites were hypermethylated covering 115 miRNAs.

Supplementary FIGURE S4: Comparison of mir-199a-1's host gene (*DNM2*) mRNA expression in HCC tumor, precursor and normal liver tissues

Significant under-expression of *DNM2* mRNA was observed in HCC tumor compared with precursor and normal liver tissues, which is consistent with the expression pattern of miR-199a in HCC tumor tissue.

Supplementary FIGURE S5: Comparisons of mRNAs in HCC tumor, precursor and normal liver tissues

A representative target gene for miR-125b (*LIN28B*) is displayed in Figure S5A. *LIN28B* was significantly over-expressed in HCC tumor compared with precursor and normal liver tissues ($p<0.001$). Figure S5B displays a representative target gene for miR-199a (*mTOR*) that was significantly over-expressed in HCC tumor compared with precursor and normal liver tissues ($p<0.001$).

Supplementary TABLE S1: Comparison of clinical and pathological characteristics for HCC subjects in the discovery (10 patients) and validation sets (56 patients)

Supplementary TABLE S2: Significantly aberrant DNA methylation of CpG sites associated with miRNAs in comparison of 10 pairs of HCC tumor and adjacent non-tumor tissues (adjusted $p<0.05$)

Supplementary TABLE S3: Validation of the 28 significant CpG sites in additional 56 pairs of HCC tumor and adjacent non-tumor tissues (adjusted $p<0.05$)

Supplementary TABLE S4: Validation of the inverse correlations between DNA methylation differences and miRNAs fold changes (N=56 pairs)

Supplementary FIGURE S1

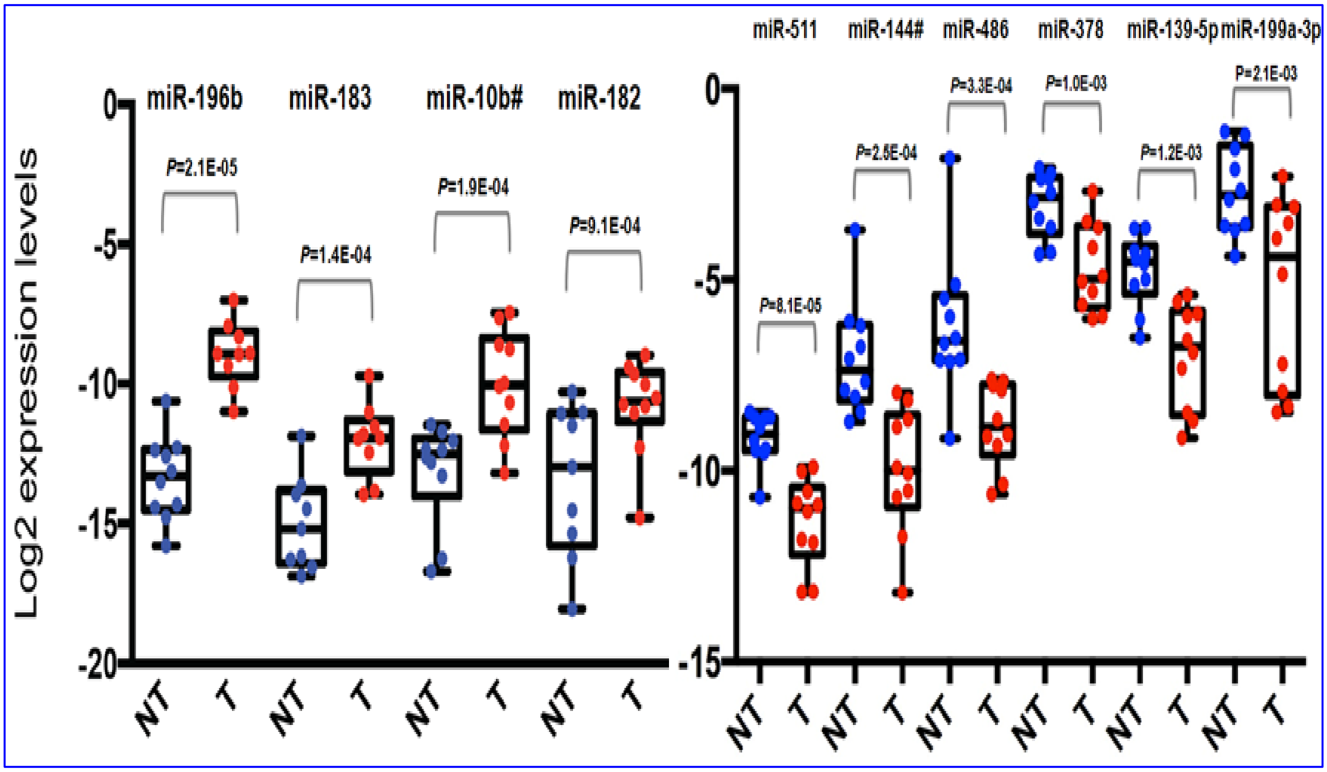

Supplementary FIGURE S2

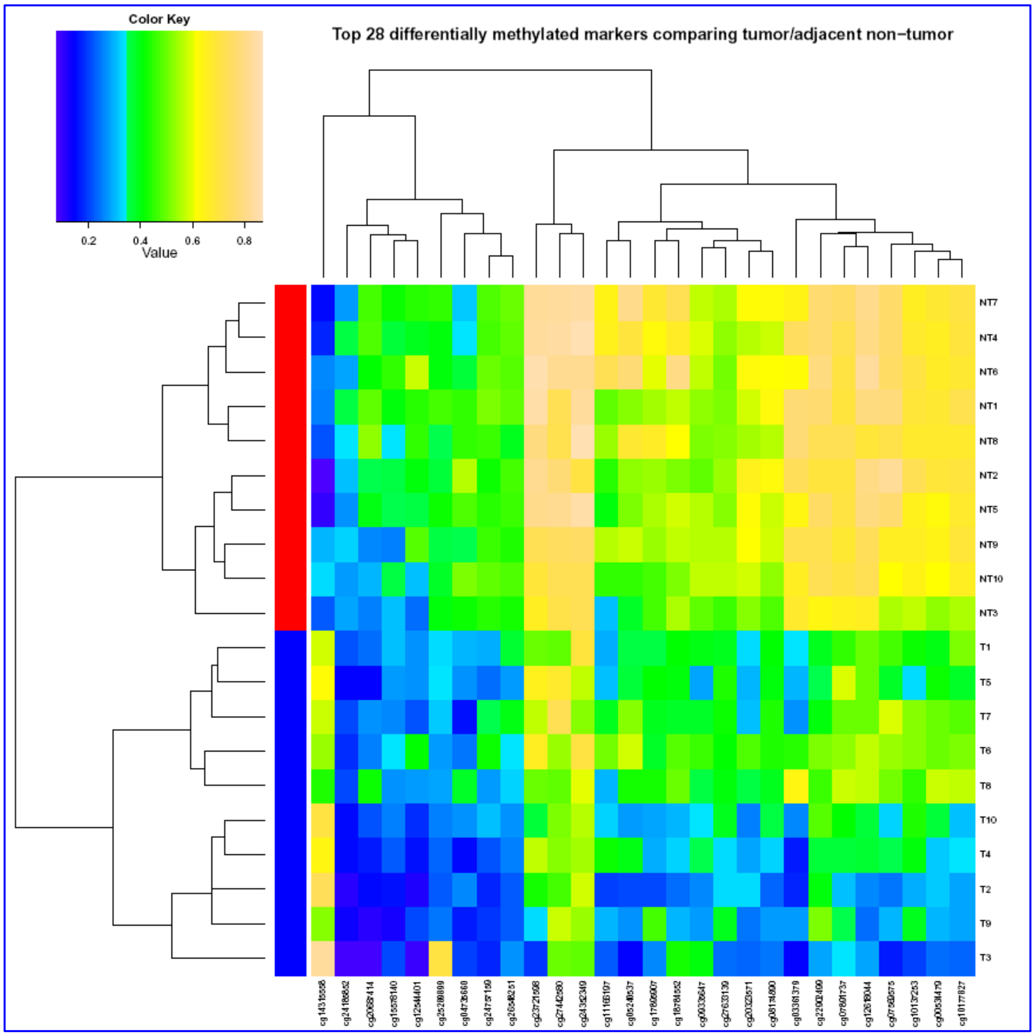

Supplementary FIGURE S3

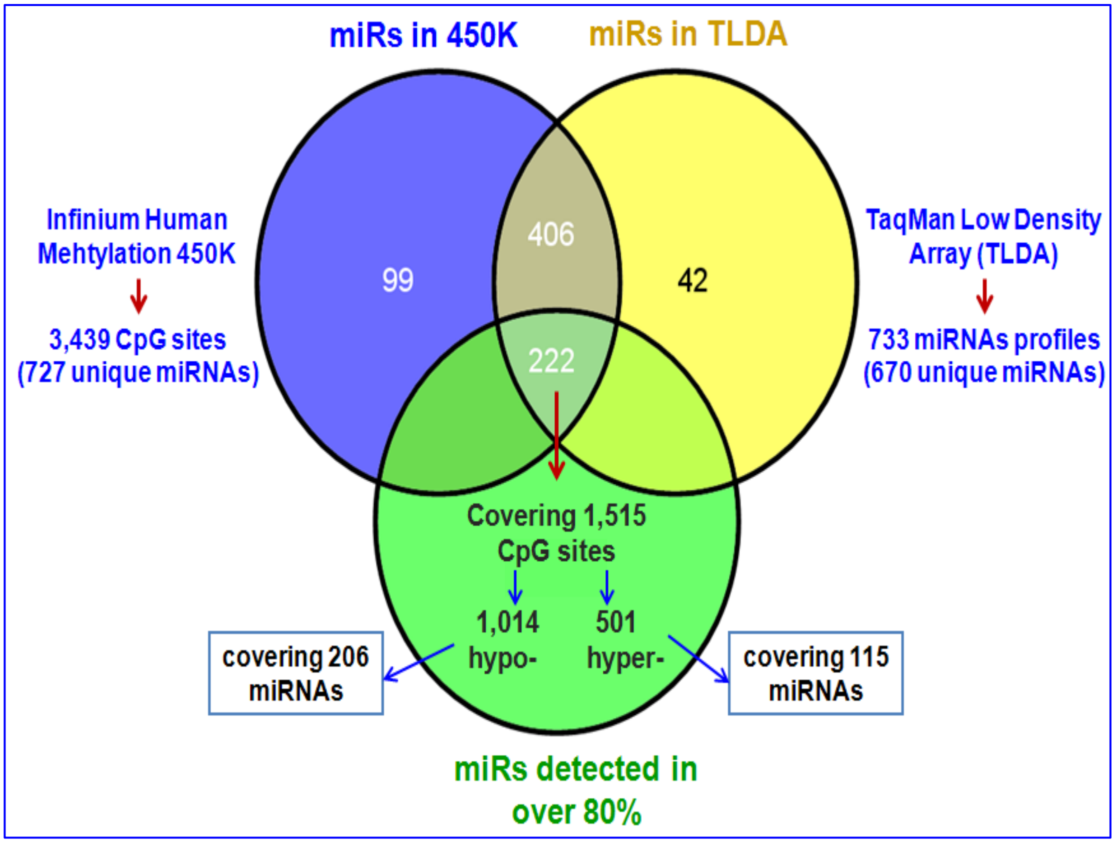

Supplementary FIGURE S4

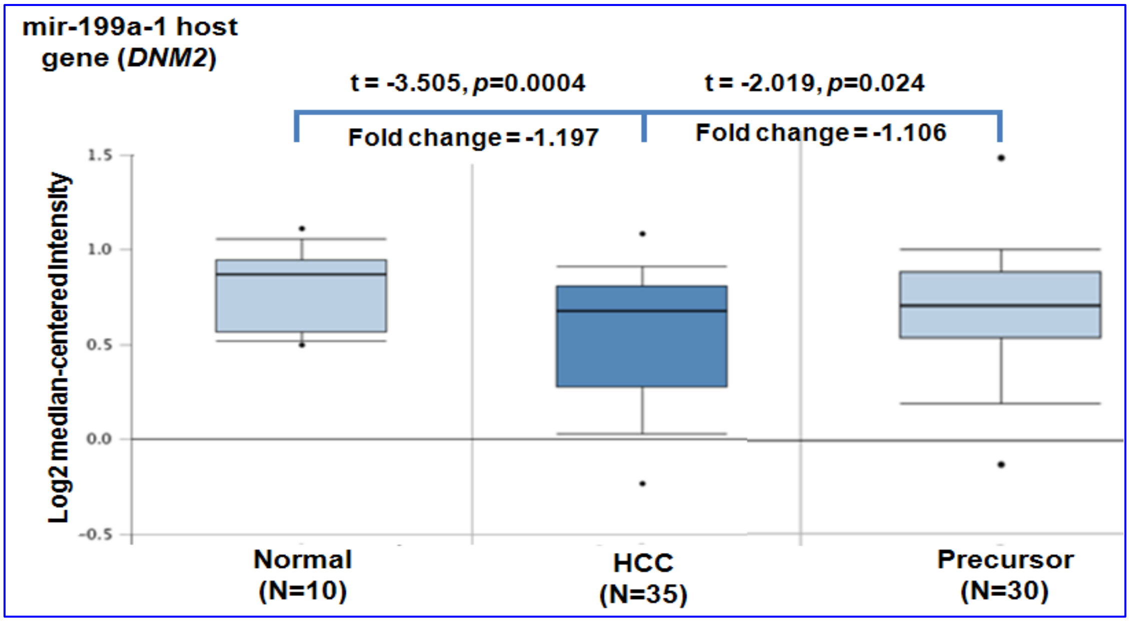

Supplementary FIGURE S5

Supplementary FIGURE S5A

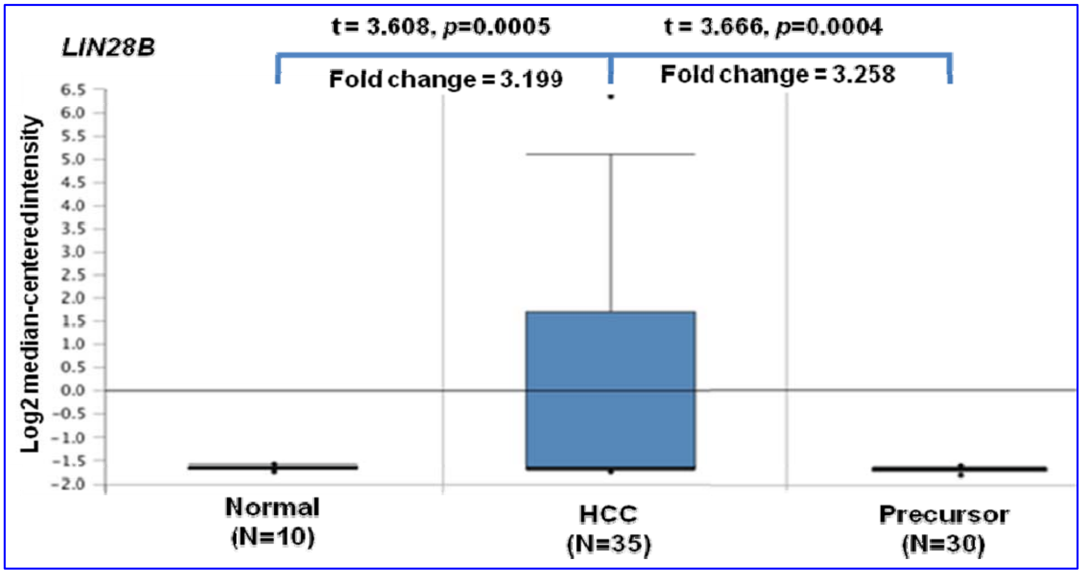

Supplementary FIGURE S5B

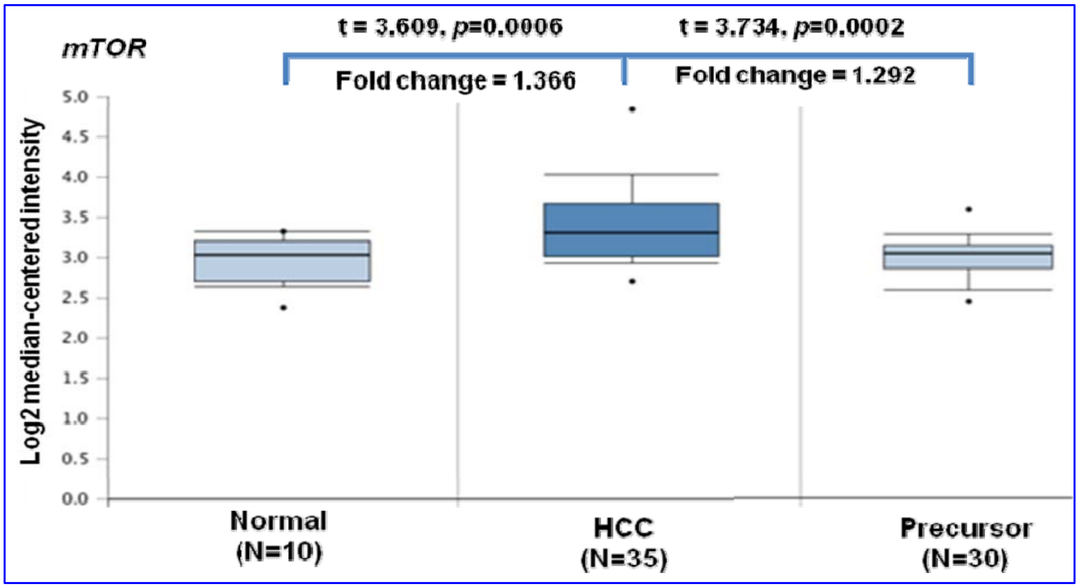

Supplementary TABLE S1: Comparison of clinical and pathological characteristics for HCC subjects in discovery set (10 patients) and validation set (56 patients)

Variables	Discovery set	Validation set	*p* values
Age at diagnosis, Mean ± SD	62.7 ± 13.1	58.9 ± 14.9	4.53.E-01
Age at diagnosis, No (%)			
< 60 yrs	3 (30.0)	26 (46.4)	
≥ 60 yrs	7 (70.0)	30 (53.6)	3.35.E-01
Gender, No (%)			
Male	5 (50.0)	45 (80.4)	
Female	5 (50.0)	11 (19.6)	**3.91.E-02**
Ethnicity, No (%)			
Caucasian	3 (30.0)	31 (55.5)	
African-American	2 (20.0)	4 (7.1)	
Hispanic	2 (20.0)	4 (7.1)	
Asian	3 (30.0)	11 (19.6)	
Unknown/Other	0 (0.0)	6 (10.7)	3.98.E-01
Viral infection, No (%)			
HBV (−), HCV (−)	1 (10.0)	18 (32.2)	
HBV (−), HCV (+)	5 (50.0)	14 (25.0)	
HBV (+), HCV (−)	4 (40.0)	9 (16.1)	
HBV (+), HCV (+)	0 (0.0)	4 (7.1)	
Missing	0 (0.0)	11 (19.6)	7.70.E-02
Cigarette smoking, No (%)			
No	6 (60.0)	21 (37.5)	
Yes	4 (40.0)	32 (57.1)	
Missing	0 (0.0)	3 (5.4)	3.60.E-01
Alcohol drinking, No (%)			
No	5 (50.0)	22 (39.3)	
Yes	5 (50.0)	31 (55.4)	
Missing	0 (0.0)	3 (5.4)	6.64.E-01
AFP (ng/mL), Mean ± SD	6086.8 ± 17237	10275 ± 52910	6.68.E-01
Tumor size (cm), Mean ± SD	7.8 ± 3.9	5.8 ± 4.8	2.12.E-01
Cirrhosis, No (%)			
No	2 (20.0)	15 (26.8)	
Yes	7 (70.0)	41 (73.2)	
Missing	1 (10.0)	0 (0.0)	5.60.E-02
Tumor grade*, No (%)			
I-II	3 (30.0)	20 (35.7)	
III	4 (40.0)	18 (32.1)	
IV	3 (30.0)	15 (26.8)	
Missing	0 (0.0)	3 (5.4)	8.48.E-01
Surgery, No (%)			
Hepatic resection alone	8 (80.0)	20 (35.7)	
Transplant alone	0 (0.0)	32 (57.1)	
Both hepatic resection and transplant	2 (20.0)	4 (7.2)	8.41.E-04
Survival, No (%)			

Yes	5 (50.0)	33 (58.9)	
No	5 (50.0)	23 (41.1)	5.99.E-01

* Edmondson and Steiner grade

Supplementary TABLE S2: Significantly aberrant DNA methylation of CpG sites associated with miRNAs in comparison of 10 pairs of HCC tumor and adjacent non-tumor tissues (adjusted *p*<0.05)

TargetID	Mean in HCC tumor tissue	Mean in HCC non-tumor tissue	Mean difference (tumor vs. non-tumor)	Adjusted P value	CHR	MAPINFO	Name of miRNAs	Host genes
cg22902499	0.4172	0.7379	-0.3207	5.44E-04	6	97754012	miR-548H3	MIR548H3
cg20323571	0.3141	0.5941	-0.2800	1.08E-03	1	176999034	miR-488	MIR488;ASTN1;ASTN1
cg05240537	0.3564	0.5961	-0.2397	2.66E-03	X	103346747	miR-1256	MIR1256;MCART6
cg24757159	0.2661	0.4685	-0.2024	8.94E-03	3	99345675	miR-548G	MIR548G
cg27633139	0.3750	0.5307	-0.1556	9.47E-03	4	9558893	miR-548i2	MIR548I2
cg25289899	0.3222	0.4020	-0.0798	9.49E-03	11	2019167	miR-675	H19;MIR675
cg24185852	0.1610	0.3099	-0.1489	9.61E-03	5	168690818	miR-585	MIR585;SLIT3
cg23721598	0.4791	0.7869	-0.3078	1.00E-02	15	79501052	miR-184	MIR184
cg27442580	0.5532	0.7720	-0.2189	1.17E-02	6	97797349	miR-548H3	MIR548H3
cg08174890	0.3533	0.5829	-0.2296	1.26E-02	4	9558077	miR-548i2	MIR548I2
cg10137253	0.3971	0.6663	-0.2692	1.82E-02	14	101507590	miR-300	MIR300
cg15578140	0.2442	0.3627	-0.1185	1.91E-02	7	147718109	miR-548F3	MIR548F3;CNTNAP2
cg11166197	0.3269	0.5248	-0.1979	1.96E-02	X	151561584	miR-105-1	GABRA3;MIR105-1
cg12618044	0.4175	0.7645	-0.3470	2.62E-02	X	103296301	miR-1256	H2BFM;MIR1256
cg04735660	0.2420	0.4271	-0.1851	2.72E-02	1	176999916	miR-488	MIR488;ASTN1;ASTN1
cg00534479	0.3945	0.6490	-0.2545	2.76E-02	5	168196464	miR-218-2	SLIT3;MIR218-2
cg12544401	0.2224	0.4123	-0.1899	2.89E-02	X	103291721	miR-1256	MIR1256
cg07891737	0.4451	0.7052	-0.2601	2.96E-02	19	54215577	miR-519D/miR-517A	MIR519D;MIR517A
cg26548251	0.2969	0.4637	-0.1667	3.36E-02	7	147591462	miR-548F3	MIR548F3;CNTNAP2
cg20687414	0.2064	0.3946	-0.1881	3.73E-02	3	44155764	miR-138-1	MIR138-1
cg09335647	0.3530	0.5481	-0.1950	3.76E-02	7	147097440	miR-548i4	MIR548I4;CNTNAP2
cg24352349	0.5830	0.7996	-0.2166	3.96E-02	22	45597767	miR-1249	C22orf9;C22orf9;MIR1249

cg10177827	0.3933	0.6765	-0.2833	3.96E-02	14	101511425	miR-487B/miR-381	MIR487B;MIR381
cg18784552	0.3780	0.6123	-0.2343	4.35E-02	X	113885574	miR-1912	MIR1912;HTR2C
cg07569575	0.3885	0.7204	-0.3319	4.44E-02	5	168691938	miR-585	SLIT3;MIR585
cg03361379	0.3041	0.6996	-0.3954	4.68E-02	7	157367159	miR-153-2	PTPRN2;PTPRN2;MIR153-2;PTPRN2
cg17895907	0.3512	0.5711	-0.2199	4.78E-02	X	146366330	miR-514-3/miR-514-2	MIR514-3;MIR514-2
cg14315558	0.6215	0.2117	0.4098	4.99E-02	21	26934682	miR-155HG	MIR155HG

Supplementary TABLE S3: Validation of the 28 significant CpG sites in additional 56 pairs of HCC tumor and adjacent non-tumor tissues (adjusted *p*<0.05)

TargetID	Mean in HCC tumor tissue	Mean in HCC non-tumor tissue	Mean difference (tumor vs. non-tumor)	Adjusted P value	CHR	MAPINFO	Name of miRNAs	Host genes
cg22902499	0.4333	0.7277	-0.2945	9.71E-17	6	97754012	miR-548H3	MIR548H3
cg20323571	0.2885	0.5780	-0.2895	9.27E-24	1	176999034	miR-488	MIR488;ASTN1;ASTN1
cg05240537	0.3728	0.6486	-0.2758	7.31E-17	X	103346747	miR-1256	MIR1256;MCART6
cg24757159	0.2874	0.4781	-0.1907	1.36E-16	3	99345675	miR-548G	MIR548G
cg27633139	0.3648	0.5513	-0.1865	2.27E-17	4	9558893	miR-548i2	MIR548I2
cg25289899	0.2865	0.4153	-0.1288	2.16E-10	11	2019167	miR-675	H19;MIR675
cg24185852	0.1734	0.3085	-0.1351	3.46E-22	5	168690818	miR-585	MIR585;SLIT3
cg23721598	0.4487	0.7566	-0.3079	1.04E-16	15	79501052	miR-184	MIR184
cg27442580	0.4493	0.7353	-0.2860	6.68E-15	6	97797349	miR-548H3	MIR548H3
cg08174890	0.3638	0.5888	-0.2250	1.90E-18	4	9558077	miR-548i2	MIR548I2
cg10137253	0.4059	0.6654	-0.2595	4.34E-19	14	101507590	miR-300	MIR300
cg15578140	0.2326	0.3489	-0.1163	2.53E-12	7	147718109	miR-548F3	MIR548F3;CNTNAP2
cg11166197	0.3728	0.5662	-0.1934	4.82E-13	X	151561584	miR-105-1	GABRA3;MIR105-1
cg12618044	0.4226	0.7702	-0.3476	9.10E-19	X	103296301	miR-1256	H2BFM;MIR1256
cg04735660	0.2641	0.4619	-0.1979	5.55E-18	1	176999916	miR-488	MIR488;ASTN1;ASTN1
cg00534479	0.3706	0.6521	-0.2815	1.10E-18	5	168196464	miR-218-2	SLIT3;MIR218-2
cg12544401	0.2452	0.4749	-0.2298	4.49E-19	X	103291721	miR-1256	MIR1256
cg07891737	0.4199	0.6837	-0.2638	1.47E-16	19	54215577	miR-519D/miR-517A	MIR519D;MIR517A
cg26548251	0.2865	0.4431	-0.1566	3.51E-11	7	147591462	miR-548F3	MIR548F3;CNTNAP2
cg20687414	0.2009	0.4088	-0.2079	5.21E-17	3	44155764	miR-138-1	MIR138-1
cg09335647	0.3079	0.5441	-0.2362	6.87E-20	7	147097440	miR-548i4	MIR548I4;CNTNAP2
cg24352349	0.6480	0.7787	-0.1307	2.57E-10	22	45597767	miR-1249	C22orf9;C22orf9;MIR1249
cg10177827	0.4204	0.6903	-0.2699	1.30E-19	14	101511425	miR-487B/miR-381	MIR487B;MIR381
cg18784552	0.3516	0.5914	-0.2398	5.67E-16	X	113885574	miR-1912	MIR1912;HTR2C

cg07569575	0.3930	0.7246	-0.3317	2.48E-19	5	168691938	miR-585	SLIT3;MIR585
cg03361379	0.3158	0.6886	-0.3729	1.61E-20	7	157367159	miR-153-2	PTPRN2;PTPRN2;MIR153-2;PTPRN2
cg17895907	0.3012	0.5452	-0.2440	1.63E-18	X	146366330	miR-514-3/miR-514-2	MIR514-3;MIR514-2
cg14315558	0.5998	0.2146	0.3852	3.11E-17	21	26934682	miR-155HG	MIR155HG

Supplementary TABLE S4: Validation of the inverse correlations between DNA methylation differences and miRNAs fold changes (N=56 pairs)

**miRNAs (chromosome location)**	**CpG sites**	**Distance to miRNA (bp)**	**Methylation difference (T vs. NT)**	**Fold change (T vs. NT)**	**Spearman Correlation Coefficients**	***p* values**
**mir-10a**	cg15649236	195	0.1	-1.45	0.04	6.69.E-01
17q21.32	cg01572694	246	0.03	-1.45	-0.03	7.26.E-01
46,657,200-46,657,309	cg14884929	347	-0.01	-1.45	0.28	**3.20.E-03**
**mir-18a**	cg17799287	1241	-0.07	1.27	-0.04	7.09.E-01
13q31.3	cg07641807	1046	-0.09	1.27	-0.06	5.17.E-01
(92,002,997-92,003,088)	cg23665802	667	-0.09	1.27	-0.01	9.26.E-01
	cg02297838	551	-0.11	1.27	-0.08	4.01.E-01
**mir-125b-1**	cg02101355	142	0.02	-1.51	-0.23	**1.35.E-02**
11q24.1	cg03891346	173	0.004	-1.51	-0.27	**3.90.E-03**
121,970,465-121,970,552	cg24150623	540	0.11	-1.51	-0.3	**1.40.E-03**
	cg06749053	667	0.08	-1.51	-0.29	**1.60.E-03**
	cg20475322	680	0.17	-1.51	-0.29	**1.80.E-03**
	cg24603444	782	0.09	-1.51	-0.27	**3.90.E-03**
	cg16865908	0	0.04	-1.51	-0.18	6.20.E-02
	cg26916936	61	0.06	-1.51	-0.1	3.04.E-01
	cg07685357	1402	0.07	-1.51	-0.29	**2.10.E-03**
**mir-182**	cg16576544	0	-0.09	2.31	-0.18	5.39.E-02
7q32.2	cg24423782	85	-0.09	2.31	-0.15	1.19.E-01
129,410,223-129,410,332	cg17677032	95	-0.04	2.31	-0.01	8.82.E-01
	cg04579608	103	-0.07	2.31	-0.11	2.66.E-01
	cg13713066	325	-0.08	2.31	-0.16	9.76.E-02
**mir-199a-1**	cg27648270	0	0.07	-2.58	-0.61	**1.00.E-04**

19p13.2<br>10,928,102-10,928,172	cg18544365	6	0.06	-2.58	-0.61	**1.00.E-04**
	cg23047544	39	0.08	-2.58	-0.61	**1.00.E-04**
	cg02660440	61	0.08	-2.58	-0.63	**1.00.E-04**
	cg06754197	150	0.04	-2.58	-0.55	**1.00.E-04**
	cg02907064	155	0.07	-2.58	-0.59	**1.00.E-04**
	cg23068797	377	0.09	-2.58	-0.61	**1.00.E-04**
	cg03216043	467	0.1	-2.58	-0.6	**1.00.E-04**
	cg13965612	524	0.07	-2.58	-0.54	**1.00.E-04**
**mir-1180**	cg04864152	15	-0.01	1.57	-0.15	1.06.E-01
	cg20272287	18	-0.02	1.57	-0.15	1.10.E-01
	cg02796621	678	-0.05	1.57	0.23	**1.54.E-02**
	cg02206323	1181	-0.05	1.57	0.23	**1.64.E-02**
	cg26619894	1277	-0.06	1.57	0.28	**2.90.E-03**
